# A Review on Roller Compaction Quality Control and Assurance Methods for Earthwork in Five Application Scenarios

**DOI:** 10.3390/ma15072610

**Published:** 2022-04-01

**Authors:** Qinglong Zhang, Zaizhan An, Zehua Huangfu, Qingbin Li

**Affiliations:** 1 Department of Civil Engineering, School of Civil and Resource Engineering, University of Science and Technology Beijing, Beijing 100083, China; qlzhang@ustb.edu.cn; 2 State Key Laboratory of Hydroscience and Engineering, Tsinghua University, Beijing 100084, China; 3 Railway Engineering Research Institute, China Academy of Railway Sciences Corporation Limited, Beijing 100081, China; azz14@tsinghua.org.cn; 4 Qianping Reservoir Construction and Management Administration, Zhengzhou 450003, China; hfzh@163.com

**Keywords:** quality control, quality assurance, earthwork, digital rolling compaction, automatic rolling compaction, intelligent control compaction

## Abstract

Successful quality control and quality assurance (QC/QA) of earthwork compaction is critical to the long-term performance of roads, railways, airports, dams, and embankments. The purpose of this paper is to provide insights into the current practice, existing problems, challenges, and future development trends of QC/QA methods from the perspective of bibliometrics and the development stage. A bibliometric analysis is presented. Through quantitative analysis of literature and qualitative analysis of the development stage, insights into the current research practices and future directions of QC/QA methods have been derived from the perspectives of literature, cluster analysis, classification, different types of QC/QA methods, conclusions, and recommendations. It is found that the current QC/QA methods can be roughly divided into conventional compaction, digital rolling compaction, automatic rolling compaction, and intelligent control compaction. Currently, QC/QA methods are mainly confronted with the issues of accurate detection of compaction quality, autonomous optimization and intelligent decision-making of compaction process, multi-machine coordination, QC/QA-related specification formulation, and process standardization. To address these issues, several critical potential research directions are further identified: comprehensive CCI measurement system; simple and realistic mathematical representation of the complex compaction dynamics; parallel computing and distributed management of multi-source heterogeneous data; standardized application workflow and the cost–benefit assessment in the context of the full life cycle; intelligent control theories, methods, and technologies of earthwork compaction based on multidisciplinary integration. The paper enables researchers to obtain a comprehensive understanding of QC/QA methods for earthwork compaction as well as the suggested solutions for future work.

## 1. Introduction

Quality control (QC) and quality assurance (QA) of earthwork compaction are critical to ensure the stability and service life of the infrastructure, such as roads [1,2,3], railways [4,5,6], airports [7,8], dams [9,10], and embankments [11,12]. The objectives in implementing QC/QA were to improve the quality of the earthwork, reduce life-cycle costs, return the responsibility of quality to the contractor, and to reduce conflicts. In recent years, the large-scale construction of infrastructure has brought huge challenges to the quality management of earthwork, because these projects require to achieve the above objectives [13,14,15]. Conventional compaction methods, which relay on prediction and simulation analysis and influencing factor analysis before construction, on-site supervision during construction, and post-compaction spot tests, have the following limitations. First, sampling tests with obvious disadvantages of point sampling and destructive measurements, low efficiency, high cost, and higher requirement for operators cannot reflect the compaction quality of the entire working area, nor can it meet the actual needs of the large-scale mechanized construction [2,10]. Second, manual supervision is prone to misjudgment, poor control accuracy, and considerable human influence [16,17]. Third, prediction and simulation analysis as well as influencing factor analysis before construction have no timeliness. In addition, conventional compaction methods may interfere with the subsequent construction operation [13].

Digital rolling compaction methods have become a solution to some of the problems associated with conventional compaction methods [18,19,20]. This type of method realizes QC/QA by continuously detecting the compaction quality and real-time monitoring of the compaction parameters. With the deepening of research and the practical application of various types of projects, several representative digital rolling compaction technologies have been proposed one after another, such as continuous compaction control (CCC) [21,22,23], roller-integrated compaction monitoring (RICM) [24,25,26,27], and intelligent compaction (IC) technologies [28,29,30,31]. So far, digital rolling compaction theory and methods have been widely used in real projects [32,33,34,35,36,37]. Although the QC/QA problems have been partially addressed by digital rolling compaction methods, there are still many problems to be solved further, such as low detection accuracy, large dispersion of detection results, complexity of interpreting the compaction status, and increased errors in the results. In addition, evident problems related to control of compaction parameters have remained unresolved, such as manual driving will lead to control error, and the operator will significantly influence the control effect of compaction parameters, thus resulting in inferior compaction uniformity.

To solve the foregoing problems, as well as deficiencies in conventional compaction methods, some researchers proposed automatic rolling compaction methods based on automatic control theory and automatic navigation and driving technology [7,38,39,40]. This method enables the roller to drive automatically, further eliminates the influence of human operation, and realizes the accuracy control of the compaction parameters. Currently, it has been used in the construction of earthwork, especially earth–rock dams [17,38,41,42,43]. Digital rolling compaction and automatic rolling compaction have overcome some of the shortcomings of conventional compaction methods, but the technical level of QC/QA for earthwork still stays at the stage of automatic control and human decision. For the existing QC/QA methods, the construction process has procedural characteristics; the compaction quality control is still completed by manual offline decision-making and evaluation; different construction areas are rolling compaction with fixed compaction parameters, which cannot be optimized independently. With the development of new non-destructive testing methods, automatic control technology, and artificial intelligence (AI) technology, problems related to existing QC/QA methods are expected to be resolved. Gradually, a new machine-material-information-machine intelligent decision-making framework is formed [17,44,45]. Based on this framework, various types of intelligent compaction control methods have been proposed by several researchers [17,46,47].

Apparently, the breakthrough and progress made in the QC/QA methods are the key to improving the management level of compaction quality in earthwork. The existing research indicates that the collaborative control of multiple unmanned rollers, high-accuracy continuous compaction detection methods, machine intelligent decision-making methods, and multiple heterogeneous data integration and management are important directions for achieving breakthroughs. To provide an overview of the state-of-the-art in the QC/QA methods, and to reveal possible challenges and future directions, this paper conducts a systematic review of relevant literature. The discussion of the paper starts with the methodology and classification for the QC/QA methods in Section 2. Section 3, Section 4, Section 5 and Section 6 present various types of QC/QA methods of various eras divided in accordance with the development stage and technical level and summarizes the existing problems of QC/QA methods. Finally, Section 7 concludes the current research efforts and discusses directions for future.

## 2. Methodology and Classification

The overall methodology for the review consists of six steps: (1) research scope is first determined and (2) the search conditions are defined on the basis of the research scope, including time span, key words, etc.; (3) and then different types of available literature are collected, and a preliminary statistical analysis is performed; again, (4) QC/QA methods are classified; (5) the development venation of QC/QA methods is statistically analyzed in detail, and the problems of existing methods in each era are discussed in detail; finally, (6) conclusions and future directions are summarized.

### 2.1. Research Scope

**Figure 1:**
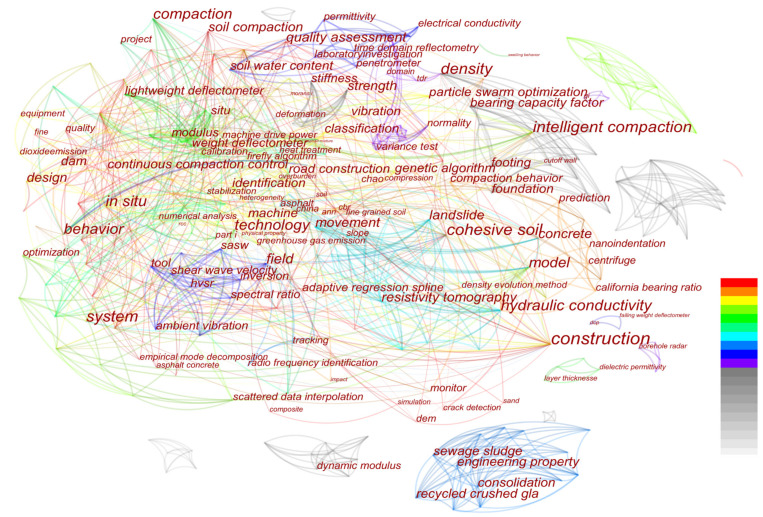
Keyword clustering.

Firstly, earthwork is the core economic and technical issue of infrastructure construction, which directly determines the quality, construction period, and investment. Ensuring the construction quality of earthwork has an extremely important role and significance for infrastructure. Secondly, compaction is an important link, and the compaction quality of earthwork has a decisive influence on the safe and stable operation of infrastructure. Therefore, the main content of the essay belongs to the research scope of compaction quality management of earthwork. Again, it is widely known that QC/QA are essential for achieving satisfactory construction quality. Quality control is specifically assigned to contractors, such as the paving contractor in subgrade and pavement construction. Quality assurance refers to actions that are necessary to accept the construction quality and to certify that the construction quality being evaluated is that which the owner indicated. Quality assurance is assigned to state agencies, such as Department of Transportation. In addition, a keywords analysis was conducted by using CiteSpace, which is a Java application for trends and pattern visualization, and demonstrated that roads and dams were the most common types of infrastructure in the analyzed papers, as shown in Figure 1. Similar to roads, earthworks in railways and airports are also crucial. Therefore, this paper will focus on QC/QA methods for earthwork compaction in five application scenarios.

### 2.2. Literature Sources and Statistics

**Figure 2:**
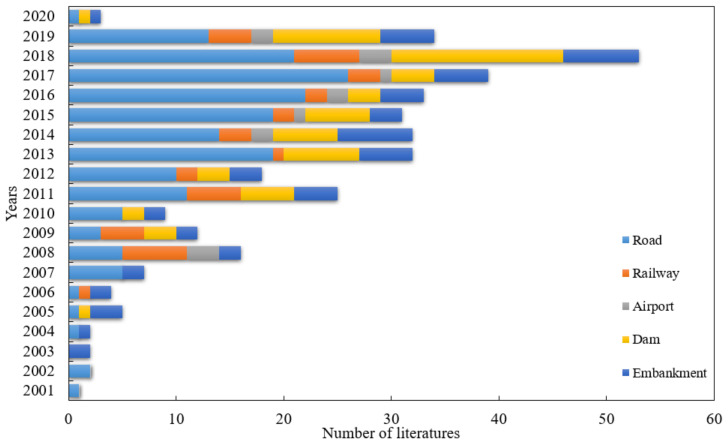
Published literature related to the QC/QA methods for earthwork.

Based on the above research scope, key words including “compaction quality road”, “compaction quality railway”, “compaction quality airport”, “compaction quality dam”, and “compaction quality embankment” are used when searching relevant papers, and web of science is taken as the main source of literature. After the above-mentioned rough search, a detailed filtering was carried out in combination with the subject of the QC/QA methods for earthwork compaction. In the light of statistics, there were 365 relevant literatures published from 1990 to 2020 (till 31 December 2020). Although the coverage may be a limitation, the database covers almost all the important publications in the field, which can reflect the current practice. For the key word “compaction quality road”, there were a total of 183 related literature, including 156 articles and 27 proceedings papers. The search and filtering of “compaction quality railway” showed that there were 39 literature, namely, 34 articles and 5 proceedings papers. There are only a few works in literature related to “compaction quality airport”. The literature on “compaction quality dam” consists of 61 articles and 8 proceedings papers. In terms of “compaction quality embankment”, there were 60 related literature works. A statistical analysis of the existing 365 literature showed that there are much more literature after 2000 than the period before 2000. Therefore, the data of relevant literature presented below are retrieved from 2001 to 2020. As in Figure 2, most of the collected literature concern the QC/QA methods of roads, followed by dams and embankments, and a small portion of them focus on railway and airport.

**Figure 3:**
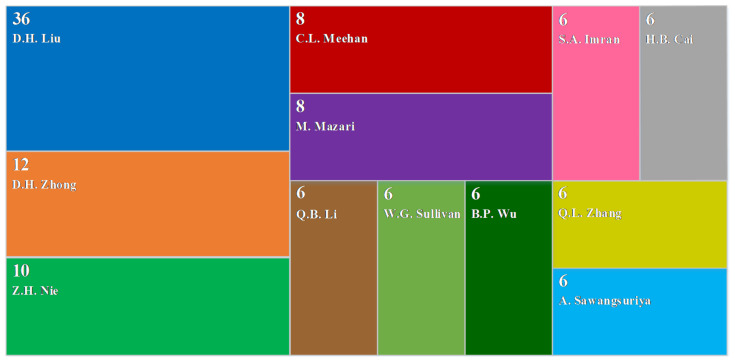
Top 10 authors in search of related literature by key words.

**Figure 4:**
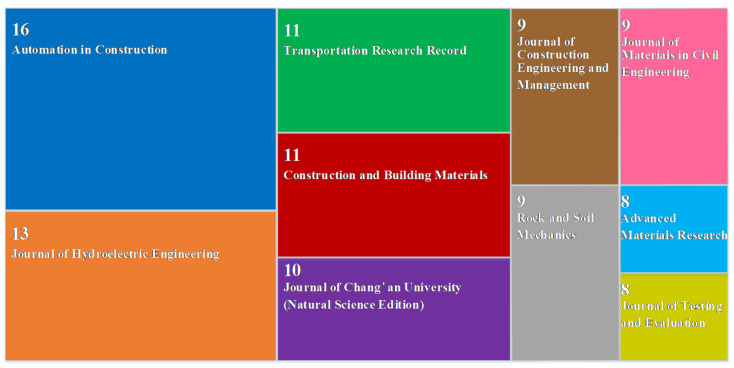
Top 10 publishers in search of related literature by key words.

**Figure 5:**
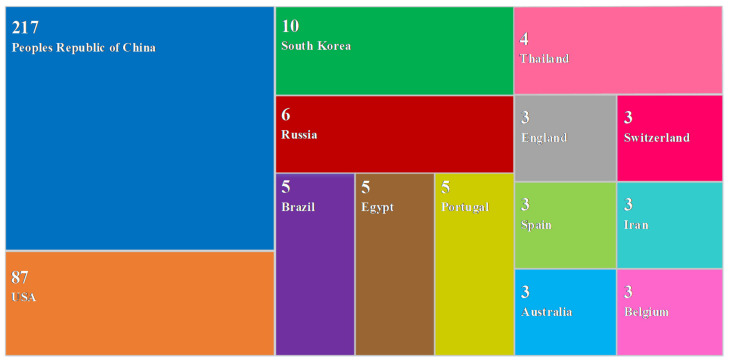
Top 10 countries in search of related literature by key words.

CiteSpace was used to perform cluster analysis on the retrieved literature; the spatial distribution of authors, journals, and countries was studied; and top 10 authors, top 10 journals, and top 10 countries related to the QC/QA methods for earthwork were extracted (Figure 3, Figure 4 and Figure 5). Then, key researchers such as D.H. Liu, etc.; key publishers including Automation in Construction, etc.; and key countries involving the People’s Republic of China, USA, etc. were identified. Figure 3 and Figure 5 show that compared to other countries and their corresponding scholars, the People’s Republic of China and the United States have more researchers and fruitful results in the study of the QC/QA methods for earthwork compaction. This is related to the continuous increase in investment in infrastructure construction in China in recent years, and the leading position of the United States in certain aspects related to the QC/QA methods. Figure 3 indicates that the current research is mainly carried out around construction technologies, filling materials, in situ tests, and indoor tests.

### 2.3. Classification of Compaction Quality Control and Assurance Methods for Earthwork

**Figure 6:**
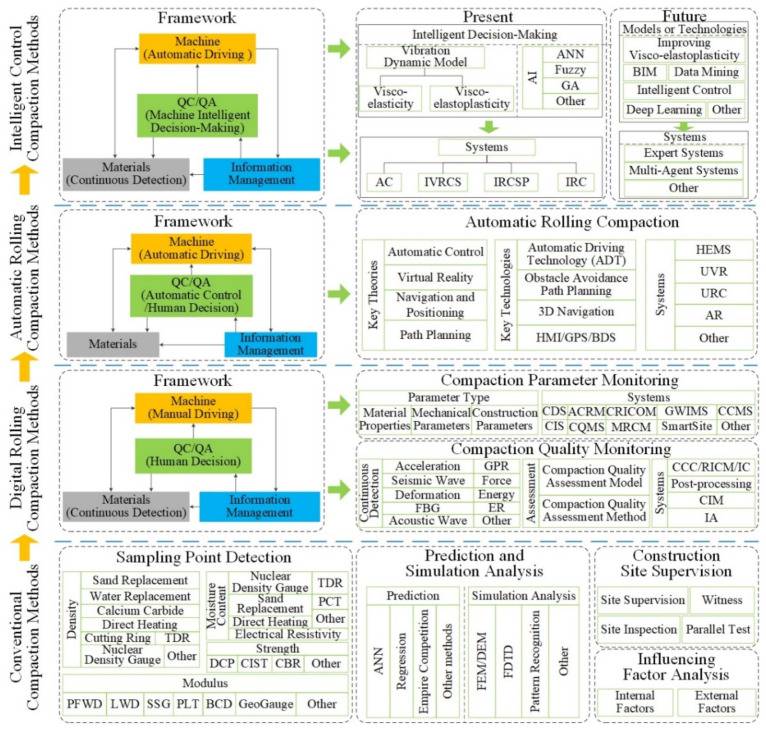
Classification of compaction quality control and assurance methods for earthwork.

With a deep reviewed and cluster analysis, a classification as illustrated in Figure 6 is presented to clarify the development venation of the QC/QA methods for earthwork compaction. Obviously, the revolutionary development stages can be roughly divided into four eras, namely conventional compaction, digital rolling compaction, automatic rolling compaction, and intelligent control compaction. In the era of conventional compaction, manual driving, manual monitoring, and sampling point detection are the salient features of this period. For the sake of achieving real-time monitoring of compaction parameters and compaction quality, the researchers proposed digital rolling compaction technology. The QC/QA methods have developed from the 1.0 era to 2.0 era marked by manual driving, digital monitoring and management, sampling point detection, and spatial positioning. With the goal of accuracy control of the compaction parameters, scholars have put forward automatic rolling compaction technology, which partially realizes the active control of the compaction process. In this era, the manual driving mode was transformed into an automatic driving mode. With the development of new technology, QC/QA problems related to existing digital rolling compaction and automatic rolling compaction are expected to be resolved. Based on the framework of material-machine-information-machine intelligent decision-making, the intelligent control compaction methods make the QC/QA methods enter the 4.0 era. In the new era, the ultimate intention is to solve present problems like comprehensively eliminating the influence of human factors on compaction quality control and verification. With this classification in mind, the following sections will discuss each part of it in detail.

## 3. Conventional Compaction Method

As an empirical control method, the conventional compaction has the characteristics of manual driving, construction site supervision, manual recording, and sampling point detection [10,17,24,44]. Currently, conventional compaction methods can be divided into four types: sampling point detection, prediction and simulation analysis, construction site supervision, and influencing factor analysis [44,48,49,50,51,52]. In actual engineering, conventional compaction methods mainly rely on the manual control of compaction parameters (such as the number of compaction times, compaction trajectory, vibration frequency, lift thickness, and driving speed) during construction, as well as the sampling point detection (such as compactness or dry density) of specified locations after construction [7,10,48,53] to ensure compaction quality of earthwork. Sampling point detection methods are diverse and widely used, and most of the methods have been written into specifications and standards [48,49,50,51,52]. Prediction and simulation analysis methods can provide a certain qualitative or quantitative description of soil-roller and soil compaction [54,55]. In a certain process or part of the construction project, the supervisor will spend all or part of the time on the construction site to track and supervise the rolling construction activities, which is the construction site supervision. In term of influencing factor analysis, it is the primary problem to be solved in constructing a compaction quality assessment model.

### 3.1. Sampling Point Detection

Conventionally, the compaction quality of filling materials suitable for earthwork is evaluated through spot detection of the density, moisture content, strength, and modulus at some discrete points [10,53]; for instance, the sand cone method [56], electromagnetic soil density gauge method [57], direct heating method [58], nuclear method [59], and water-filling method [60] are currently used for detecting the density. As moisture content go, the methods mainly include the nuclear method [59], sand cone method [56], and direct heating method [58]. The dynamic cone penetrometer (DCP) method [61] and Clegg impact soil test (CIST) method [62] are used to detect strength. In terms of modulus, the lightweight deflectometer (LWD) method [63], soil stiffness gauge (SSG) method [64], and plate loading test (PLT) method [65] are detection methods in common use. Take the sand cone method as an example—it is a commonly used test method in the subgrade, and suitable for in situ determination of the density and moisture content of fine-grained soil, sand soil, and gravel soil [56,66]. Up to now, the sampling point detection methods have been widely used in roads, railways, airports, dams, and embankment. These methods have high detection accuracy and have become the benchmark method for other methods. However, there are the following main problems that need to be further resolved: (1) the compaction status of the entire working area cannot be effectively reflected; (2) most methods are destructive, which greatly disturbs the compaction area; (3) low efficiency and high cost severely restrict the construction schedule, reduce construction efficiency, and project economics; (4) partial methods have high requirements for operators and operation accuracy; (5) the compaction quality of the entire working area cannot be recorded in real time, and data traceability is extremely deficient.

### 3.2. Prediction and Simulation Analysis

In terms of prediction methods, researchers have achieved certain research results. Since the soil–roller interaction exhibits complex properties such as elastic-plasticity and non-linearity, and the stress distribution of the soil profile is extremely uneven, it is difficult to describe the compaction state of soil qualitatively or quantitatively. In the research of many scholars, it is mentioned that when an average ground pressure is given when the wheels have more load, the deeper places tent to produce tighter compaction [67,68,69]. For soil compaction, there are also some documents mentioning that tire parameters and wheel loads have significant meaning [70,71]. Klos and Waszczyszyn [72] used neural networks to predict the compaction characteristics of coarse-grained soils. Patel and Mani [54] conducted an on-site survey on sandy loam to determine the compaction of the subsoil under different ranges of wheel loads, multiple passes through the bulk density foundation, and multiple penetration resistance indicators. Through experiments, Raper and Reeves [73] evaluated the different between soil bulk density and cone index (CI, soil hardness) obtained under various conditions such as topsoil plowing, subsoil deep plowing, and fixed track tillage. Çarman [74] utilized the Mamdani fuzzy logic method to study the compaction of clay loam under conditions of pneumatic tires with different travel speeds, different wheel loads and inflation pressures, and believed that artificial neural network research is necessary for the further development of the system. Bayat et al. [75] compared the three methods of ANN, linear regression, and non-linear regression, used to predict penetration resistance, and demonstrated that ANN has the advantage of higher accuracy for multiple linear regression methods. Taghavifar et al. [76] proposed an optimization algorithm of hybrid artificial neural network and empire competition algorithm for predicting soil compaction, which has good qualitative and quantitative analysis accuracy. Cosanti et al. [77] proposed an innovative method to evaluate the compactness of river embankments.

A lot of progress has also been made in simulation analysis. Li and Schindler [78] used the finite element method (FEM) to analyze soil compaction and tire fluidity, developed two finite element tire models based on the real geometry of Bridgestone tires, and analyzed the influence of axle load and tire pressure on soil compaction. Based on the viscoelasticity of the soil, Zolotarevskaya [79] deduced the regression equation as a function of its density, moisture content, and linear compaction speed, and performed mathematical simulations and calculations on the compaction of the soil under dynamic load conditions. Shoaib and Kari [80] used the discrete element method (DEM) to perform a nonlinear elastoplastic shock wave simulation for high-speed compaction. Ghanbari and Hamidi [81] conducted a simulation analysis on the rapid impact compaction process of loose sand. Xia [82] established an FEM that can simulate the compaction process of the soil and predict the spatial density. Simulation analysis based on this model shows that the proposed large-deformation FEM can flexibly predict the compaction density of the soil. Shangguan et al. [83] presented the application of ANN-based pattern recognition to extract the density information of asphalt pavement from simulated GPR signals.

In general, the prediction methods are mainly based on ANN, linear regression, and non-linear regression to predict the compaction characteristics and compaction situation of soil. Some scholars also used CCC/RICM/IC technology to predict the compaction of the filling materials and interpolated the compactness of the entire work area. The simulation analysis methods typically utilize FEM and DEM to carry out mathematical simulation and calculation analysis on the compaction characteristics of soil under dynamic load conditions. Although the prediction and simulation analysis can provide a certain qualitative or quantitative description of soil–roller interaction as well as soil compaction, it is not time-sensitive, and cannot effectively control and manage compaction quality in real time.

### 3.3. Construction Site Supervision

Construction site supervision is usually used to judge whether the compaction quality during construction meets the design requirements [48,49,50,51,52,84]. Supervisors implement project supervision by means of site supervision, witness, site inspection, and parallel test [85,86,87]. The purpose of construction site supervision is to urge the contractor to strictly follow the relevant national laws and regulations, contractual agreements, design documents, and construction specifications to carry out the project construction, to ensure that the non-conforming problems in the construction of the project can be corrected and resolved in time, thereby ensuring achievement of supervision goals. Site supervision is to ensure that the key procedures or key operations meet the requirements of the specification. It embodies the process control, but it is by no means a “single station” for the supervision. Witness is a supervision activity that supervisors can see with their own eyes and can testify, and its essence is the control of key points by the supervisors. Site inspection is the most common and largest supervision method, which focuses on understanding the situation and discovering problems. Parallel test is an activity carried out by the supervisors to conform whether the performance of the project inspection item is qualified, and its essence is the re-examination of the construction quality. Site supervision, witness, site inspection, and parallel test are the four most basic methods of construction site supervision, which is a systematic method structure. For QC/QA of earthwork, construction site supervision is an important and indispensable link.

### 3.4. Influencing Factor Analysis

There are many factors in the construction of earthwork that affect its compaction effect. For the sake of ensuring the strong stability and strength of the earthwork, it is necessary to identify and analyze the main factors affecting the compaction effect, and then take targeted control measures to obtain a better compaction effect. Zhang [88] discussed several problems in QC/QA of earth–rock dam, and found that the type of soil, moisture content, and compaction energy have a greater impact on the compaction effect of the soil during construction. Guo [89] analyzed the influence of various factors such as compaction machinery, driving speed, moisture content, strength of the underlying layer, and rolling mode on the compactness of the pavement. Based on the surface vibration compaction method, Guo [90] carried out an indoor compaction test for natural gravel and found that the main factors affecting its compaction effect were the rock content, gradation, and vibration parameters. By monitoring the compaction parameters such as driving speed, the number of compaction times, lift thickness, and the state of the exciting force, Liu et al. [91] evaluated the factors that affect the compaction effect of earth–rock dam. For the soil–rock mixture, Tokiharu et al. [92] used the method of compaction test to study the change of the maximum dry density of the material with the maximum particle size, and the results showed that the two have a linear correlation. In the light of rockfill materials and soil–rock mixtures, Sitharam and Nimbkar [93] carried out research on the effects of different gradations and volume strains on the properties of granular materials. Liu and She [94] carried out compaction tests for different types of soil–rock mixtures, which showed that the compactness of soil–rock mixtures is closely related to compaction energy and compaction methods.

Summarizing the above research, the factors that affect the compaction effect of earthwork can be divided into internal factors and external factors. Specifically, the internal factors mainly include material properties, material type, and the strength of the foundation or underlying layer; the external factors mainly consist of compaction energy, compaction parameters, rolling machinery, compaction mode, and compaction strategy. Under the same compaction energy, the properties or types of materials determining the compaction characteristics of the filling materials are not the same. When other conditions are roughly the same, the main influence on the compaction effect is the moisture content of the materials. If the strength of the underlying layer or foundation is insufficient, the compaction effect of the filling layer will be half the effort. The compaction energy directly affects the compaction quality and construction efficiency of the earthwork, and the appropriate compaction energy will greatly improve the construction efficiency and economy. Other influencing factors attributable to internal or external factors have a greater or lesser effect on QC/QA of earthwork. Regardless of internal or external factors, it is indispensable to analyze the influencing factors related to QC/QA of earthwork.

## 4. Digital Rolling Compaction Method

According to existing specifications, QC/QA of earthwork mainly depends on the control of compaction parameters (the number of compaction times, compaction trajectory, vibration frequency, lift thickness, and driving speed) during construction and random spot tests after construction [48,49,50,51,52]. To achieve real-time monitoring of compaction parameters and compaction quality, researchers have proposed digital rolling compaction methods and technologies, such as continuous compaction control (CCC) [21,22], roller-integrated compaction monitoring (RICM) [3,15], and intelligent compaction (IC) [29,30,31]. The digital rolling compaction method is implemented based on the framework of material-machine-information-manual decision-making. Since QC/QA of earthwork are closely related to compaction parameters and compaction quality, the research of digital rolling compaction methods mainly focuses on monitoring compaction parameters and compaction quality (see Figure 6). Combined with the cluster analysis results in Figure 1, digital rolling compaction methods are the current research hotspot in the QC/QA methods of earthwork and have been widely used in the construction of roads [11,23,32], railways [34,95], airports [8,35], and dams [24,36,96].

### 4.1. Compaction Parameter Monitoring

The compaction parameter monitoring first determines the compaction parameters through the on-site compaction test, and then realizes the real-time monitoring of compaction parameters through the RTK base station, satellite, master control center, and GPS positioning equipment installed on the roller. In 1989, Thurner and Sandstrom [97] developed a compaction documentation system for unbound aggregates. Pampagnin et al. [98] proposed an architecture of a GPS-based guiding system and exploited a computer integrated road construction of compaction (CIRCOM). To explore the relationship between vibration characteristics and underlying soil properties, Mooney and Rinehart [99] carried out field monitoring study on roller vibration during compaction of subgrade soil. Immediately afterwards, they developed a comprehensive instrumentation system (CIS) to monitor the three-dimensional vibration of roller compactor components and the phase lag of the drum response with respect to the eccentric force [100]. In addition to CIRCOM, other researchers have also conducted compaction parameter monitoring research for QC/QA of road construction and have successively developed compactor tracking system (CTS), pavement compaction process monitoring system (PCPMS), remote intelligent monitoring system (RIMS), and intelligent compaction monitoring system (ICMS). As for earth–rock dams, most research focuses on the monitoring of mechanical parameters and construction parameters during compaction. Some systems [101,102,103,104,105,106] have been developed to improve the compaction quality and construction efficiency of earth–rock dams, such as compaction quality monitoring system based on the positioning compensation technology (CQMS), Georobot/WLAN-based intelligent monitoring system (GWIMS), real-time supervisory system (RTSS), continuous compaction monitoring system (CCMS), and compaction quality monitoring system for rockfill dam (RDCQMS). For ensuring compaction efficiency of earth–rock dam construction, Liu et al. [101] developed the automatic control and real-time monitoring system (ACRM) to realize the automatic control of the moisture content of earth–rock dam material. Liu et al. [102] created a cyber-physical system for collaborative control of multiple rollers (MRCM). In addition, several researchers have carried out compaction parameter monitoring studies on embankments [107], high filled channels [108], and earthworks [100], and developed compaction equipment instrumented with global positioning system technology (GEGPS), monitoring and control system of roller compaction quality (CQMCS), and Comprehensive instrumentation system (CIS), respectively.

**Figure 7:**
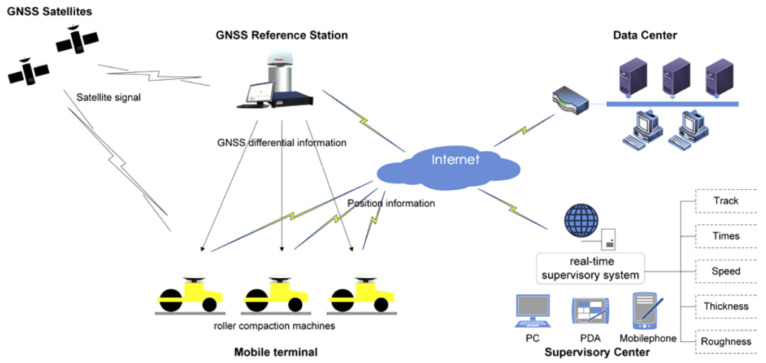
The GNSS real-time compaction quality supervisory system (reprinted with permission from Reference [105], copyright Elsevier, 2018).

Combining the existing specifications related to QC/QA of earthwork [48] as well as the existing research related to compaction parameter monitoring (see Table 1), the monitored compaction parameters can be roughly divided into three types, namely, material properties, mechanical parameters, and construction parameters. Among them, material properties mainly refer to moisture content and particle gradation; mechanical parameters mainly include excitation force, vibration frequency, and amplitude; construction parameters mainly consist of lift thickness, the number of compaction times (see Figure 7), compaction trajectory, driving speed, the control standards of moisture content, and the amount of water added to the filling materials. Focusing on the monitoring of different compaction parameters, researchers have developed various types of compaction parameter monitoring systems using computer technology, automatic control technology, communication technology, three-dimensional modeling technology, and GPS/BDS positioning technology, with the aim of improving compaction quality and construction efficiency. The existing compaction parameter monitoring methods and systems still rely on the driver to realize the operation of the roller, and the compaction quality of earthwork is still seriously affected by human factors. Although the compaction parameters can be monitored in real time, the active and accuracy control of the compaction parameters cannot be achieved, and there are problems such as rolling omission, cross rolling, and repeated rolling around the joint of two adjacent layers.

### 4.2. Compaction Quality Monitoring

Compaction quality monitoring provides a new solution to part of the problems associated with conventional compaction methods [10,16]. Studies on the compaction quality monitoring have primarily focused on the continuous detection method, compaction quality assessment method, and compaction quality monitoring system (see Figure 6). As the core of continuous detection, the continuous compaction index (CCI) is used to characterize the compaction quality of the filling materials [67]. Based on CCI, some researchers have established different types of compaction quality assessment models, and further proposed corresponding compaction quality assessment methods [10,24]. For realizing a comprehensive, real-time, on-line, automatic monitoring of compaction quality, the compaction quality monitoring system integrated with CCI or compaction quality assessment method was introduced. Currently, various types of compaction quality monitoring systems have been widely used in earthwork, particularly in the quality control of road construction [3,14,15,29,30,110,111,112,113,114]. With the popularization and application of compaction quality monitoring technology in earthwork, problems associated with the conventional compaction methods have been partially addressed.

#### 4.2.1. Continuous Detection Method

Continuous detection of compaction quality is the key to realizing compaction quality monitoring of earthwork. With the development of the economy and the construction of earthwork, various types of continuous detection methods for compaction quality have been put forward in succession [115,116,117,118,119,120,121,122,123,124,125,126,127,128,129,130,131,132,133,134,135,136,137,138,139,140,141,142,143,144,145,146,147,148,149,150,151,152,153,154,155,156,157,158,159,160,161,162,163,164,165,166,167,168,169,170,171,172,173,174,175,176,177,178,179,180,181,182], such as the acceleration method, GPR method, seismic wave (SW) method, force method, deformation method, energy method, FBG method, acoustic wave method, electrical resistivity (ER) method, and other methods (see Table 2). Currently, the above-mentioned continuous detection method has been applied to varying degrees in various scenarios, and these scenarios mainly include five types of road, railway, airport, dam, and embankment. As shown in Table 2, the acceleration method, GPR method, and surface wave method have been successfully applied in the five main application scenarios. Based on the measured acceleration, the researchers proposed different CCIs, namely, CMV [1,16,23,25,37,97,112,115,127,129,133,147,149,151,161,162,163,164,167,168,169,170,171,181], RMV [112,118,162,172], CCV [2,133,147,172], CV [24,91,122,124,125,130,172], CF [131], $A_{p}$ [119], OMV [138], AA [147], $F_{t}$ [166], and THD [100,123,172,175]. The GPR method is mainly used for testing the moisture content and compactness of subgrade, thickness detection of structural layer, investigation of road damage, and void identification [4,113,139,140,152,173,174].

Since the propagation speed of seismic waves is different in various media, the compaction quality of soil can be estimated by establishing the relationship model between wave speed and soil density. The researchers applied the seismic wave method to continuously detect the compaction quality of earthwork. In conformity with the propagation mode, the seismic wave method can be divided into three types: longitudinal wave (P wave), transverse wave (S wave), and surface wave (L wave). Therefore, the seismic wave method can be divided into P wave method, S wave method, and surface wave method. As of right now, the surface wave method has application records in the main application scenarios [6,120,128,143,154,156,157,158,160,173,174,176,178]. The P wave method has successively served the construction of roads [155], airports [177], and dams [117,142]. Similarly, the applicability of S wave on roads [141], airports [177], and dams [142] has also been verified. In terms of force method, Xu et al. [12,34,126,132,133] proposed a structural resistance detection method based on VCV index, which characterizes the changes in compaction state of highway subgrade, railway subgrade, and embankment through changes in the resistance of the filling material to compaction machine. In addition, Gao [125] constructed a foundation reaction index considering the lag phase angle to characterize the compaction quality of the earth–rock dam.

Both modulus and stiffness are closely related to the elastic deformation of the materials. Therefore, CCI of the modulus or stiffness type is classified as a deformation method. The CCI used in this method is mainly $k_{s}$ [30,99,125,161], $E_{vib}$ [111,144,145,146,161], and $M_{i}$ [114,135,136,137]. Deformation methods based on these indexes have been explored and studied in road, railway, and airport. Here, the method of using the change of energy to reflect the change of the compaction state during compaction is classified as the energy method. So far, there are mainly five types of CCI in the energy method, namely MDP [1,23,25,147,149,150,151,162,163,182], Omega [111,161], E [16,123], DMV [121], CEV [168]. The application scenarios of the energy method based on these indexes are scattered in the construction of road, railway, airport, dam, and embankment. In addition, there are many other methods used to detect the compaction quality of the filling materials in the earthwork. These methods provide a variety of options for the QC/QA of earthwork. Based on fiber bragg grating (FBG) technique, Chen et al. [165] introduced a FBG method for soil subgrade vertical deformation monitoring. For filling materials of the earth–rock dam with a large particle size distribution, an acoustic wave detection method is proposed, and the corresponding sound compaction value (SCV) adopted by the engineering construction [10,17,36]. For the electrical resistivity method, several studies related to the detection of compaction quality of soil or other filling materials have been carried out [116,179,180]. Besides the above methods and CCIs, a soil stiffness measurement method combined with contact stress distribution (CSD) and a normalized compaction indicator (NCI) as function of soil stiffness have been introduced to enrich the continuous detection method of earthwork.

Existing continuous detection methods and CCIs are widely used in roads and dams, while the application research in railways, airports, and embankments needs to be strengthened. The above research shows that the acceleration method and corresponding CCIs are applicable to the compaction quality detection of fine-grained soil, coarse-grained soil, and giant-grained soil. In the meantime, practical engineering applications also highlight that the main problems of this type of method are the large discrete data of CCIs, low detection accuracy, and poor robustness. The GPR method is mainly used to detect the moisture content and compactness of subgrade and other fillings, the compactness of pavement, the thickness of the structure layer, the investment of pavement damage, and the identification of voids. This type of method has high detection efficiency and belongs to the non-destructive detection method, but its accuracy is not enough, the data processing is complicated, and it is difficult to meet the needs of earthwork in all application scenarios (Table 2). Since the propagation speed of waves is different in different media, the compactness of soil can be estimated by establishing the relationship model between wave speed and soil density. The SW method is suitable for detecting the compactness of fine-grained soil, coarse-grained soil, and giant-grained soil. This method is efficient and convenient and does not disturb the filled soil itself. However, due to the diverse and complex nature of the soil, the propagation of waves in different types of soil is not the same and mechanism has not been deepened. In addition, the measurement results have large errors, and multiple calibrations are required before use, which requires high operators and is quite difficult to promote. Obviously, the most urgent problem to be solved for other types of methods is to expand the scope of application to different types of soil, and to strengthen their popularization and application in the earthwork.

#### 4.2.2. Compaction Quality Assessment

Although the continuous detection method based on CCI has partially solved the problems of conventional compaction methods, the impact of material source parameters, physical parameters, compaction parameters, and other parameters (such as meteorological parameters) on compaction quality and the variability of factors affecting compaction quality of earthwork still need to be further resolved. Material source parameters mainly consist of moisture content, gradation, P5 content, curvature coefficient, and uneven coefficient [10,24]. The compaction parameters mainly include the number of compaction times, compaction trajectory, driving speed, lift thickness, and vibration frequency [10,24]. Therefore, it is necessary to consider the physical parameters of the material to be crushed in the compaction quality assessment model. To reflect the compaction status of the entire work area more objectively, reasonably, and effectively, several researchers focus on compaction quality assessment models considering material source parameters, physical parameters, compaction parameters, and other parameters [183,184,185,186,187,188,189,190,191,192,193,194,195,196,197]. At present, the commonly used compaction quality assessment models mainly include regression models [10,24,123,183,188,190], neural network models [37], models based on kernel methods [37,184,185,186], fuzzy control models [129,187], and other types of models [189,191,194]. Among them, the regression model is divided into simple linear regression (SLR), simple nonlinear regression (SNR), multiple linear regression (MLR), and multiple nonlinear regression (MNR). Neural network models mainly consist of the radial basis function (RBF) neural network model, the artificial neural network (ANN) model, and a model based on bidirectional extreme learning machine (B-ELM).

Based on the above models, several compaction quality assessment methods suitable for different scenarios and materials have been proposed. The research of Nie et al. [26] showed that the combination of δ and R is capable of comprehensively evaluating filed compaction quality. Based on geostatistics with different CCIs, several researchers have proposed a variety of compaction quality assessment methods suitable for dams [10,24] and roads [124]. Wang et al. [191] applied a method of detecting concept drift and updating the compaction quality assessment model to assess compaction quality of earth–rock dam. With support vector regression (SVR) with chaotic firefly algorithm (CFA) as the core, Liu et al. [184] and Wang et al. [185] constructed a comprehensive evaluation method through a combination of the improved analytic hierarchy process (i-AHP) method and the improved geo-statistical analysis method (i-GAM), and an assessment method based on SVR with CFA, respectively. On this basis, Wang et al. [186] proposed a compaction quality assessment method combining smart bacteria-foraging algorithm-based customized kernel support vector regression (SBFA-CKSVR) and enhanced probabilistic neural network (EPNN). In addition to the above assessment methods, a small number of researchers have put forward several real-time assessment methods of compaction quality, such as fuzzy evaluation method based Dempster-Shafer (DS) evidence theory. A comparison of existing compaction quality assessment models and methods is shown in Table 3. It can be seen from the table that the research on compaction quality assessment models and methods is currently mainly focused on dam construction. In addition, most existing compaction quality assessment models and methods generally do not have real-time evaluation functions, that is, they are not time-sensitive.

#### 4.2.3. Compaction Quality Monitoring Systems

**Figure 8:**
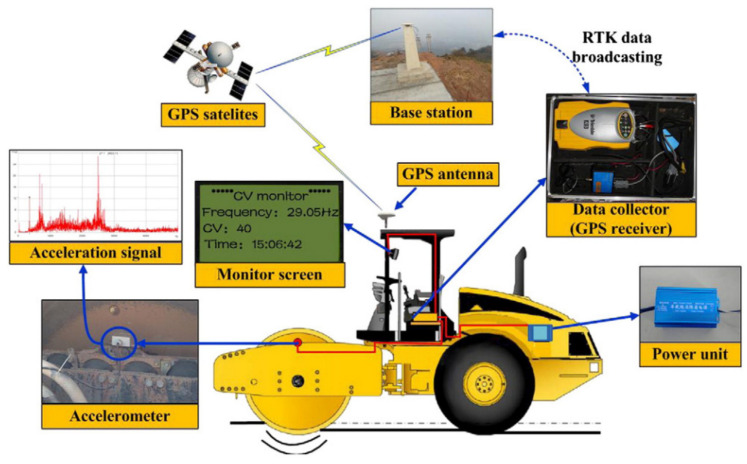
Installation of the roller-integrated compaction status monitoring device (reprinted with permission from Reference [24], copyright Elsevier, 2014).

Compaction quality monitoring systems (CQMSs) provide a new solution to the problems associated with conventional control methods [10,16,24], and a typical compaction quality monitoring system is shown in Figure 8 [24]. Studies on the CQMSs [18,103] have primarily focused on monitoring the compaction parameters [38,107,198] and the continuous detection of compaction quality [1,10,16,183]. With the development of technology and research, several typical CQMSs have matured, such as CCC/RICM/IC systems [3,15,21,22,29,30], compaction information management (CIM) systems [2,16,199], intelligent analysis (IA) systems [114,135,136,137], and post-processing systems [24,33,200,201]. The CCC/RICM system is a data acquisition system that has been installed on the compaction equipment to continuously collect real-time information regarding the detection of compaction quality and compaction parameters [15,21,202]. When the CCC/RICM system provides automatic feedback control for vibration amplitude/vibration frequency or driving speed, it is referred to as an IC system [3]. Currently, several CCC/RICM/IC systems from different manufacturers are available, including AccuGrade developed by Caterpillar and Trimble [203], ACE-Plus developed by Ammann [110], DCA developed by Dynapac [16], Aithon MT developed by Sakai [15], and BCM05 developed by BOMAG [11]. In the CCC/RICM/IC systems, the typical continuous compaction indexes used to characterize the compaction quality are CMV [25], CCV [111], Omega [162], MDP [150], $E_{vib}$ [18], $k_{s}$ [204], THD [99], and SCV [10,36]. Since the CCC/RICM/IC systems integrated with CCI were introduced, they have been widely used in earthwork, particularly in the quality control of road construction [3,14,15,29,30,110,111].

Based on the concept of information management and collaborative control, the CIM systems can be customized according to the characteristics of specific application scenarios and user needs. These systems not only provide real-time compaction information for roller operators, but also provide a collaborative basis for parties related to rolling operations (such as owners, construction personnel, and supervisors) through information integration and information sharing, with the aim of realizing multi-party Co-construction. So far, typical CIMs consist of the real-time data processing and informatics synchronization (RDPIS) system [16], SmartSite system [199], and AFC system [2]. The IA systems use artificial intelligent algorithms to build an intelligent analyzer to estimate the stiffness of the filling materials. For example, ICA [137] and IACA [205] could be used to monitor the compaction quality in real time and identify under-compacted regions during the construction of subgrade and asphalt pavement. Up to now, most IA systems are still under development. The post-processing systems takes the compaction quality assessment models and methods as the core to realize the comprehensive statistical analysis of the continuous detection data collected in the entire work area, which removes some obstacles for large-scale mechanized construction. The integration of this type of system with other types of CQMS will effectively improve its timeliness and greatly promote the large-scale application of CQMS in earthwork. Typical post-processing systems include SCAN [200], Veda [201], CMS [33], and CV-RICM [24]. Although the CQMSs mentioned above have been widely used in real projects recently, the use of such CQMSs for monitoring the compaction quality of filling materials has several disadvantages, such as low detection accuracy, large dispersion of detection results, complexity of interpreting the compaction status, increased errors in the results, being easily influenced by material factors, misjudgment and human error, and construction quality problems caused by manual driving.

## 5. Automatic Rolling Compaction Method

**Figure 9:**
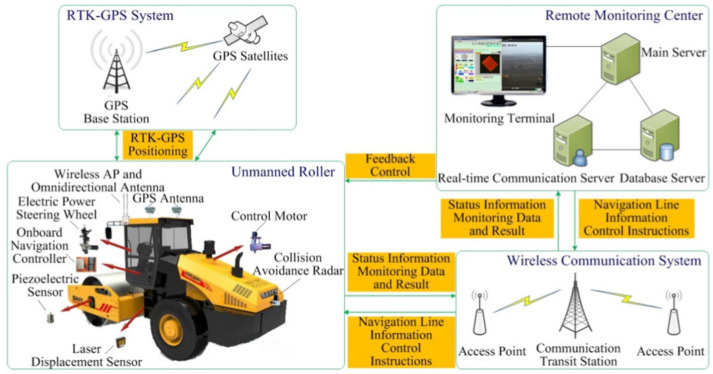
Framework of the URC system (reprinted with permission from Reference [38], copyright Elsevier, 2019).

To solve the problems caused by manual driving, some researchers have attempted to utilize automatic control technology and other key technologies (e.g., path planning) for rolling operations, and successively proposed various types of automatic rolling compaction methods [38,206,207,208,209,210,211,212,213,214,215], so as to further eliminate the influence of human factors on QC/QA. In the 1980s, Japan first applied road rolling machinery equipped with automatic control devices and rolling driving (AR) methods to asphalt pavement construction [206]. With the development of theory and technology, different automatic rolling compaction methods and unmanned rolling compaction systems have been used in roads, railways, dams, airports, and other infrastructure. Huang et al. [213] designed and implemented a PLC-based autonomous construction system of unmanned vibratory roller (UVR) for road construction. Zou et al. [215] validated a method of obstacle detection based on D-S evidence theory. For decreasing driver fatigue, an accurate trajectory tracking with disturbance-resistant was discussed [207]. To move towards high-quality road construction, autonomous tandem rollers for asphalt compaction optimization were evaluated [210]. In the field of airport engineering, a high-embankment monitoring system (HEMS) for compaction of high embankment in airport engineering was studied, which mainly includes optimal path algorithm and unmanned vehicle control technology [7,208,209]. Zhang et al. [17,38,212] carried out systematic research on the accurate control of compaction parameters, and the developed unmanned rolling compaction (URC) system was successfully applied to the construction of earth–rock dams (see Figure 9). Other researchers focused on the path tracking control and automatic driving of the unmanned vibratory roller for earth–rock dam construction [39,40,41,211,214]. In the collaborative operation of multiple unmanned rollers, Shi et al. [42] conducted a preliminary exploration and application research. As for the roller compacted concrete (RCC) dam, Shi [43] conducted an exploratory study on the implementation and application of unmanned roller compacted dam construction technology.

Several automatic rolling compaction methods and unmanned rolling compaction systems have emerged for earthwork in roads, railways, airports, dams, and embankment construction (see Table 4). These methods and systems are implemented based on the framework of material-machine-information-manual decision-making (see Figure 6). The key theories involved in realizing automatic rolling compaction include automatic control, virtual reality, navigation and positioning, and path planning. The key technologies involved in the unmanned rolling compaction system include automatic driving technology, obstacle avoidance and path planning, three-dimensional (3D) navigation, human machine interface (HMI), and GPS/BDS positioning technology. Some of them have the functions of path planning (PP), obstacle avoidance (OA), and collaborative rolling (CR). Although the automatic rolling compaction technology has partially overcome the shortcomings of the traditional rolling compaction technology, the technical level remains at the stage of automatic feedback control. Engineering applications show that the automatic rolling compaction method has the following problems: (1) the construction is still performed in accordance with the prescribed quality standards and parameters; (2) the construction process has the characteristics of step-by-step procedures; (3) the QC/QA is still completed by manual offline decision-making assessment; (4) different construction areas are rolled with fixed compaction parameters; (5) compaction parameters cannot be optimized independently.

## 6. Intelligent Control Compaction Method

Based on the cluster analysis of CiteSpace and the above review, the intelligent control compaction methods are similar to the digital rolling compaction methods, which are another research hotspot in the QC/QA methods of earthwork. With reference to the concept and principle of intelligent control in control theory, scholars have proposed different types of intelligent control compaction (ICC) methods within the framework of the material-machine-information-machine intelligent decision-making interaction system (see Figure 6). In these methods, a controller (or system) is designed to have the functions of learning, abstraction, reasoning, decision-making, etc., and to make adaptive responses according to changes in environmental information, to achieve autonomous completion of rolling operation without human intervention. ICC methods have three notable features, namely, intelligent perception of compaction information, intelligent decision-making of the working parameters, and unmanned rolling compaction. Compared with digital rolling compaction and automation rolling compaction, ICC can effectively solve compaction control problems with uncertain mathematical models, and highly non-linear and complex task requirements.

The key point of intelligent decision-making is the control criteria of the compaction parameters. Through an in-depth review of the collected papers, ICC methods can be roughly divided into three types due to the different ways of establishing control criteria [17,216,217,218]. One is a physical model-based, and the type uses a vibration compaction model simulating the vibration compaction process to optimize the working parameters of the roller [219,220,221]. Based on these simulations, the optimization of the working parameters can be conducted. The other is a mathematical model-based, and these predictive models based on artificial intelligence (AI) algorithm have attempted to adaptively adjust the working parameters to optimize the compaction process in another way [17,222]. Combining the advantages of the above two models, the third compound model aims to better improve the compaction quality and construction efficiency.

### 6.1. Vibration Compaction Model

The basic idea of the vibration compaction model is to accurately simulate the vibration compaction process, calculate the dynamic response of the soil–roller interactions, and determine the elastic–plastic deformation of the filling materials and the influence of various parameters on the compaction effect, as well as other factors. Through a great deal of vibration compaction tests, some scholars have found that the compaction effect is related to the working parameters of the roller [219,220,221]. To further understand the mechanism of vibration compaction, relevant scholars have studied the vibratory roller–soil interaction system, and established vibration compaction models to analyze the influence of different compaction parameters on the dynamic characteristics and compaction effects of the interactive system, to provide a theoretical basis for the optimization of working parameters [219,223]. Currently, the vibration compaction model can be divided into the viscoelastic model and the viscoelastic plastic model. The former includes a linear elastic model and a nonlinear elastic model, and the latter includes an asymmetric hysteretic model and viscoelastic model containing plastic components.

#### 6.1.1. Viscoelastic Model

(1) Linear Elastic Model

**Figure 10:**
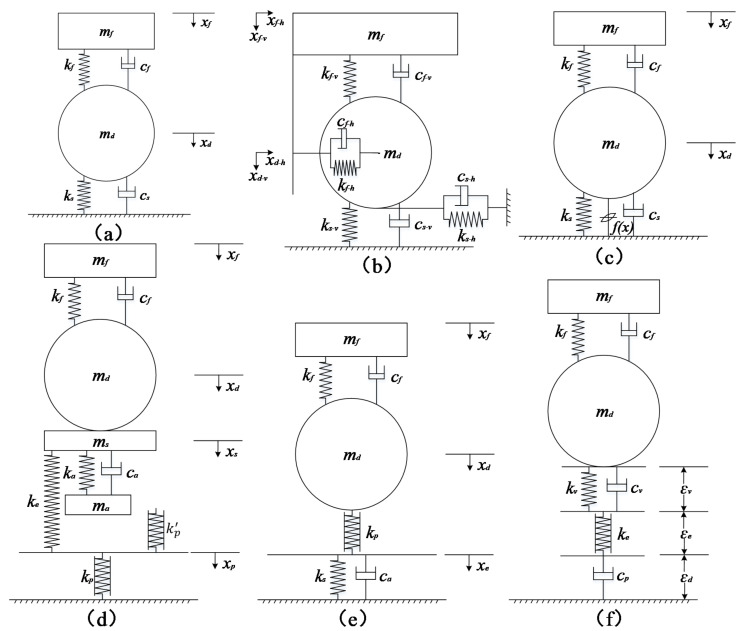
Several typical vibration compaction models. (**a**) piecewise linear model considering jumping vibration; (**b**) overall dynamic model; (**c**) asymmetric hysteresis model; (**d**) 4-DOF viscoelastic plastic model; (**e**) 3-DOF viscoelastic plastic model; (**f**) viscoelastic plastic model based on Burgers model.

**The two-degree-of-freedom (2-DOF) model**: In the 1970s, Yoo and Selig [222] proposed a classic 2-DOF vibration compaction model. The model is based on the linear elastic vibration theory and uses the mass-spring-damping system to describe the vibratory roller–soil system. As shown in Figure 10a ($k_{s}$ is the elastic stiffness of the soil; $c_{s}$ is the damping of the soil; $k_{f}$ is the stiffness of the shock absorber; $c_{f}$ is the damping of the shock absorber; $x_{f}$ is the displacement of the upper frame; $x_{d}$ is the displacement of the vibratory wheel), the model simplifies the roller into two parts, namely, the upper frame and the vibratory wheel. The two parts are connected by a shock absorber; the soil is supposed to a completely elastic body, and the Kelvin model in which a linear spring and a damper are connected in parallel is used to represent the properties of the soil.

The dynamic differential equation of the model is expressed as:

(1) $$\left[\begin{array}{cc} m_{f} & 0\\ 0 & m_{d} \end{array}\right]\left[\begin{array}{c} {\ddot{x}}_{f}\\ {\ddot{x}}_{d} \end{array}\right]+\left[\begin{array}{cc} c_{f} & -c_{f}\\ -c_{f} & c_{f}+c_{d} \end{array}\right]\left[\begin{array}{c} {\dot{x}}_{f}\\ {\dot{x}}_{d} \end{array}\right]+\left[\begin{array}{cc} k_{f} & -k_{f}\\ -k_{f} & k_{f}+k_{d} \end{array}\right]\left[\begin{array}{c} x_{f}\\ x_{d} \end{array}\right]+\left[\begin{array}{c} m_{f}\\ m_{d} \end{array}\right]=\left[\begin{array}{c} 0\\ F_{0}\sin\left(\omega t\right) \end{array}\right]$$

where $m_{f}$ is the mass of the upper frame; $m_{d}$ is the mass of the vibrating wheel; ${\dot{x}}_{f}$ and ${\ddot{x}}_{f}$ are the speed and acceleration of the upper frame, respectively; ${\dot{x}}_{d}$ and ${\ddot{x}}_{d}$ are the speed and acceleration of the vibratory wheel, respectively; $F_{0}$ is the exciting force; ω is the rotational speed of the eccentric mass; t is time.

By inputting different model parameters, the influence of the structural parameters (the distribution of the quality of the upper and lower parts, the stiffness and damping of the damper), the operating parameters (amplitude and frequency), and the soil parameters (stiffness and damping) on the dynamic response of the vibratory roller–soil system can be analyzed. The soil parameters can be identified by monitoring the dynamic response of the vibratory wheel [219,224]. The model has a simple structure, few parameters, and clear meaning, and has been widely used to analyze the interaction between the roller and the soil. It is of guiding significance for the monitoring of soil compaction status, the optimization of compaction process parameters, and the structural design of the roller. However, the model is too simplified, and some of its assumptions are inconsistent with the actual compaction situation, such as the complete elasticity of the soil and the constant contact between the vibratory wheel and the soil. Therefore, scholars later established various vibration compaction models based on it to describe the vibratory roller–soil system more comprehensively and accurately. Based on several assumptions, Fang et al. [39] established a 2-DOF vibration dynamic model, and then developed a dynamic model of the vibratory roller under the influence of exciting force. Mooney and Rinehart [99] found a 2-DOF lumped parameter vibration theory with a viscoelastic soil model to be a good predictor of roller–soil response, apart from a slightly underpredicting phase lag.

**Oscillating compaction model**: Different from the vibratory roller which mainly uses vertical vibration for compaction, in the 1980s, a new compaction technology realized compaction by applying horizontal rubbing force to the soil; the corresponding compaction machine is an oscillating roller. The mechanism of the oscillating roller is different from that of the vibratory roller, and the vibration compaction model is not suitable for the oscillating roller. For the oscillating roller, Thurner [225] proposed a single-degree-of-freedom oscillating compaction model. The model uses horizontal stiffness and damping to simulate the interaction between the oscillating wheel and the soil; the dynamic equation is shown below:

(2) $$\frac{J_{0}}{r^{2}}\ddot{x}+c_{s}\dot{x}+k_{s}x=Fsin\omega t$$

where $J_{0}$ is the moment of inertia around the center of the oscillating wheel; r is the radius of the oscillating wheel; $x,\dot{x},\ddot{x}$ are the horizontal displacement, velocity, and acceleration of the oscillating wheel, respectively.

Paulmichl et al. [153] established a lumped parameter model with 3-DOF of the interacting oscillation roller–subsoil system. With the continuous development of compaction theory and compaction machinery, the vibratory and oscillating roller appeared. The simple vibration model or oscillating model cannot meet the research needs, so the overall dynamic model (see Figure 10b) is proposed [226,227]. Considering both vertical vibration and horizontal vibration, the model can better evaluate and study the overall performance of the vibratory and oscillating roller.

**Overall dynamic model**: The 2-DOF model simplifies the roller to an upper frame-vibratory wheel system, but in fact the roller is a complex multi-degree-of-freedom mechanism. Therefore, some scholars have put forward several multi-degree-of-freedom models considering the entire vehicle of the roller, such as the 3-DOF vibration reduction system model [228], 5-DOF model [229], 6-DOF model [230], and 7-DOF model [231]. The above model not only pays attention to the interaction between the roller and the soil, but also focuses on the operating performance of the roller, such as the vibration reduction performance and driving comfort of the roller. Like the above-mentioned 2-DOF or oscillating model, the overall dynamic model generally uses a linear spring-damper system to represent the properties of the soil and describe the interaction between the vibratory wheel and the soil. In addition, this type of model provides a more detailed description of the roller that belongs to the multi-degree-of-freedom vibration body. Although this type of mode can provide guidance for the reasonable design and production of the overall structure of the roller, the model is complex and cannot effectively improve the simulation accuracy of the vibration roller–soil interaction and soil compaction effect.

(2) Nonlinear Elastic Model

**Piecewise linear model**: When the stiffness of the soil is large, the vibratory roller may separate from the soil during the rolling process, that is, the phenomenon of “jumping vibration” [232,233]. After the separation, the vibratory wheel does not interact with the soil, so the linear elastic model needs to be corrected. The rolling state is divided into two parts: contact and separation, and the piecewise linear model is established [234].

The dynamic equation of the piecewise linear model considering jumping vibration is expressed as:

(3) $$\left\{\begin{array}{c} F_{s}=-m_{d}{\ddot{x}}_{d}+k_{f}\left(x_{d}-x_{f}\right)+c_{f}\left({\dot{x}}_{d}-{\dot{x}}_{f}\right)+m_{d}g+F_{0}sin\omega t\\ -m_{f}{\ddot{x}}_{f}+k_{f}\left(x_{f}-x_{d}\right)+c_{f}\left({\dot{x}}_{f}-{\dot{x}}_{d}\right)+m_{f}g=0 \end{array}\right.$$

where $F_{s}$ is the force of vibratory wheel–soil interaction, when the vibratory wheel is in contact with the soil, $F_{s}=k_{s}x_{d}+c_{s}{\dot{x}}_{d}$; when the vibratory wheel separates from the soil, $F_{s}=0$.

**Model considering strain softening**: Experiments show that the shear modulus of soil decreases with the increase of shear strain, while the stiffness of soil is related to damping and shear modulus. Therefore, the stiffness and damping of soil are not linear, but related to strain. Wersäll et al. [235] established a vibration compaction model considering the characteristics of soil strain softening based on the 2-DOF model and proposed an iterative procedure for calculating strain-related stiffness and damping. Compared with the linear elastic model, the calculation results of this model are more consistent with the test results of a small indoor vertical vibration compactor.

#### 6.1.2. Viscoelastic Plastic Model

Assuming the soil is completely elastic and does not undergo plastic deformation, the viscoelastic model is suitable for situations that have been completely compacted or nearly completely compacted. During compaction, the soil will undergo plastic deformation from loose to compact. The viscoelastic model is not suitable for uncompacted soil, nor can it be used to analyze the compaction deformation process of soil. To describe the dynamic characteristics and the compaction deformation process more realistically, some scholars have proposed a vibration compaction model that considers plastic deformation. In accordance with the different thinking of considering the influence of plastic deformation, this type of model is mainly divided into two types: one is to add plastic deformation elements to the vibration compaction model to establish a viscoelastic–plastic model; the other is to use an asymmetric hysteretic model to describe different loading and unloading stiffness of the soil caused by plastic deformation.

**Asymmetric hysteresis model**: The soil will produce elastoplastic deformation when loading, but the elastic rebound only occurs when unloading, so the force–deformation relationship has nonlinear hysteresis characteristics. For the sake of considering this asymmetry, some scholars proposed to describe the force–deformation relationship of the soil with an asymmetric hysteretic curve and established an asymmetric hysteretic vibration compaction model (see Figure 10c).

The system dynamic equation of this model takes the following form:

(4) $$\left[\begin{array}{cc} m_{f} & 0\\ 0 & m_{d} \end{array}\right]\left[\begin{array}{c} {\ddot{x}}_{f}\\ {\ddot{x}}_{d} \end{array}\right]+\left[\begin{array}{cc} c_{f} & -c_{f}\\ -c_{f} & c_{f}+c_{d} \end{array}\right]\left[\begin{array}{c} {\dot{x}}_{f}\\ {\dot{x}}_{d} \end{array}\right]+\left[\begin{array}{cc} k_{f} & -k_{f}\\ -k_{f} & k_{f}+k_{d} \end{array}\right]\left[\begin{array}{c} x_{f}\\ x_{d} \end{array}\right]+\left[\begin{array}{c} m_{f}\\ m_{d} \end{array}\right]+\left[\begin{array}{c} 0\\ f\left(x\right) \end{array}\right]=\left[\begin{array}{c} 0\\ F_{0}\sin\left(\omega t\right) \end{array}\right]$$

where $f\left(x\right)$ is the nonlinear hysteresis force.

Grade proposed a triangular hysteretic model. Shen et al. [86] used the Bouc–Wen hysteresis model to describe the hysteresis characteristics of soil. The hysteresis curve is divided into four parts by four split displacement points ($x_{0}\sim x_{3}$), and each part has a different $f\left(x\right)$ expression. In addition, other scholars have modified or simplified these two hysteresis models, and proposed hysteresis models with different specific hysteresis curve functions [236,237,238,239]. Compared with the linear elastic model, the asymmetric hysteretic model considers the different mechanical properties of the soil during the loading and unloading process and is more in line with the dynamic characteristics in the actual compaction process. However, the model needs to set the displacements at multiple split points (or yield points), the displacements corresponding to different compaction degrees are different, and the model parameters are not easy to determine.

**Viscoelastic–plastic model with plastic components**: In the 1990s, Pietzsch et al. [240] proposed a 4-DOF viscoelastic-plastic vibration compaction model. The model consists of a roller dynamics analysis sub-module and a soil property proton module (see Figure 10d), in which $m_{f},\;m_{d},\;m_{s}$, and $m_{a}$ are the mass of upper frame, vibratory wheel, vibrating soil, and additional soil, respectively; $k_{f},\;k_{e},\;k_{p},\;k_{a}$, and $k_{p}^{'}$ are shock absorber, soil elasticity, soil plasticity, additional soil elasticity, and additional soil plastic stiffness, respectively; $c_{f}$ and $c_{a}$ are shock absorber and additional soil damping, respectively. On the basis of the contact between the vibratory wheel and the ground as well as the deformation of the soil, the model is divided into different modes, such as contact-jumping and elastic–elastoplastic.

The 4-DOF viscoelastic-plastic model can describe the dynamic characteristics and elastoplastic deformation characteristics of the soil during compaction, which is more reasonable than the viscoelastic model. For the sake of making the most effective compacting operation of intelligent vibratory roller under different compaction stages, Gong et al. [241] established a 4-DOF dynamic model of vibrating wheel-soil body under the coupling operating condition. Due to the numerous parameters of the model, especially the mass, stiffness, and damping of the vibrational soil and the additional soil are not easy to determine, the model is rarely used in practice. To make the vibration compaction model more practical, some scholars have proposed 3-DOF viscoelastic-plastic model (see Figure 10e) [231,242,243]. This model can be regarded as a simplification of the 4-DOF viscoelastic-plastic model. The linear plastic stiffness $k_{p}$ is used to describe the plastic characteristics of the soil. Elastic–plastic deformation occurs when loading, and only elastic deformation occurs when unloading.

To better understand the elastoplastic characteristics of the soil during compaction, the plastic parameter ε is defined according to:

(5) $$\varepsilon =\frac{k_{p}}{k_{p}+k_{s}}$$

The value of ε ranges is from 0 to 1. When ε is 0, it corresponds to $k_{p}=0$, and the soil is completely plastic; when ε is 1, it corresponds to $k_{p}\to \infty$, and the soil is completely plastic. Therefore, ε can reflect the compactness of the soil. Adam et al. [243] conducted a study on the change of ε with the number of compaction times. The results show that as the compaction progresses, the compactness of the soil increases, the plastic deformation decreases, the plastic stiffness increases, and ε gradually increases and tends to be stable. During simulation, different ε values can be used to correspond to the soil in different compaction stages.

Beainy et al. [217,218,244] proposed a viscoelastic–plastic compaction model (see Figure 10f), which uses the Burgers model to describe the deformation characteristics of asphalt materials. They believe that the deformation of asphalt materials when subjected to compressive strength can be divided into three parts: viscoelastic deformation, instantaneous elastic deformation, and plastic deformation. The viscoelastic deformation is represented by a parallel spring and damper, the instantaneous elastic deformation is represented by an elastic spring, and the plastic deformation is represented by a viscous damper. The parameters of the model are obtained through laboratory tests.

**Figure 11:**
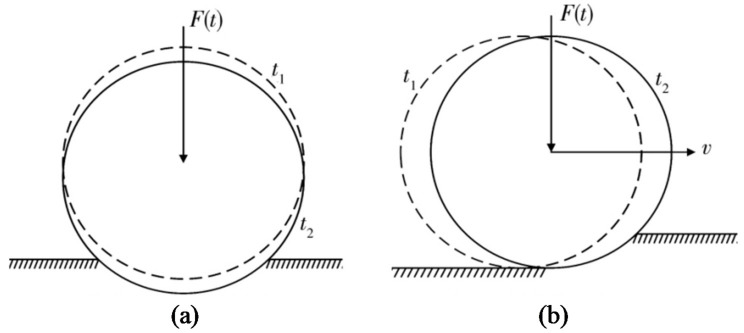
Vibration compaction in its original location and vibration compaction during driving. (**a**) vibration compaction model considering plastic deformation; (**b**) vibration compaction model considering plastic deformation and the walking of the roller.

The above vibration compaction model considering plastic deformation does not consider the walking of the roller, and its simulation is alike in the static loading shown in Figure 11a, that is, the vibratory wheel vibrates in place, and the soil under the wheel accumulates plastic deformation with time. In fact, with the continuous progress of the roller, the soil is always separated from the vibratory wheel, and the new soil is in contact (see Figure 11b). The force-displacement relationship of the vibratory wheel on the soil is different from the static loading. This type of model mainly analyzes the impact of amplitude and frequency on the dynamic response as well as compaction effect of the vibratory roller–soil system. However, it cannot analyze the impact of vehicle speed.

Imran [245] proposed a visco-elastic-plastic block model based on the visco-elastic-plastic vibration compaction model, so that the simulation process is closer to the real vibration compaction process. According to the driving direction of the roller, the model divides the contact area between the vibratory wheel and the asphalt material into a rear block, a center block, and a front block. Inside each block is Burgers material. The model assumes that the vibratory wheel is always in contact with three blocks, and the vibratory wheel–soil interaction force is the sum of the forces of the three blocks.

**Figure 12:**
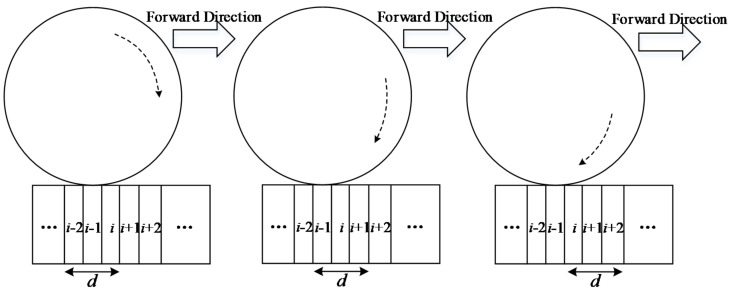
Vibration compaction model considering the walking of the roller.

On this basis, Imran [245] proposed the vibration compaction model considering the walking of the roller (see Figure 12). The pavement is evenly divided into several blocks, and the width of each block is d⁄3. When the roller is moving forward, each strip passes through the front, directly below, and behind the contact area between the vibratory wheel and soil in turn. The time of each stage is determined by the vehicle speed and wheel contact width. The block deformation can be obtained through the integration of strain over time according to these three stages. The initial condition of the integration of each stage is the integration result of the previous stage. Based on the above processing, the model can consider the impact of the roller on vibratory wheel–soil interaction as well as the impact of vehicle speed on the compaction effect. Compared with the field test, the results show that the model can effectively simulate the interaction between the roller and the asphalt pavement as well as the deformation characteristics of the asphalt.

The above research shows that the vibration compaction model used for QC/QA of earthwork has made rich progress. Research and practice have shown that the existing vibration compaction models cannot consider the elastoplastic deformation of the filling materials as well as the changes of model parameters and cannot be intelligently adjusted to the optimal compaction parameters according to the compaction situation.

### 6.2. Prediction Model Based on AI

Other models have attempted to adaptively adjust the compaction parameters using a predictive model based on AI algorithms to optimize the compaction process in another way. After determining the main influence factors on compaction parameters, Li [246] proposed a neural network model for intelligent rollers. The model consists of a prediction network and control network. The prediction network determines the maximal compactness for the next pass based on the system state variables. Next, the control network out-puts the frequency, amplitude, and roller speed. Zhang and Goh [247,248] used the back propagation neural network and multivariate adaptive regression splines to assess the pile drivability in relation to the prediction of the maximum compressive stresses, maxi-mum tensile stresses, and blow per foot. The results can be used for the optimization of dynamic compaction of HP piles. Zhang et al. [249] proposed a fuzzy control strategy for optimization of compaction parameters. The fuzzy-control rules were established from on-site data and optimized via a genetic algorithm (GA). Wang et al. [186] aimed to man-age the compaction quality assessment and control of earth–rock dams by developing a three-dimensional, real-time monitoring system based on a smart bacteria-foraging, algorithm-based, customized kernel-support vector regression, and enhanced probabilistic neural network. Isik and Ozden [250], as well as Günaydin [251] presented ANN prediction models for estimating the soil compaction parameters. Ranasinghe et al. [252,253] proposed a new and unique predictive tool developed through ANNs to predict the effectiveness of rolling compaction and improve ground compaction capability. Taghavifar et al. [76] used a hybridized ANN and imperialist competitive algorithm to predict soil compaction. Furthermore, Xu and Chang [2] developed an innovative integrated material-machine-information and human-decision system, which can provide feedback to construction personnel to optimize compaction efforts and adaptive control quality of the pavement material. Although research in the prediction model based on AI has made some progress in the optimization of QC/QA of earthwork, this model has many problems that need to be solved. Currently, relevant research is primarily focused on road construction, and there is a lack of AI algorithms to optimize the compaction process for railway, airport, dam, and embankment. There has been no effective integration and fusion study for the continuous detection of compaction quality and accurate control of compaction parameters. Moreover, the prediction model based on AI cannot accurately reflect the compaction process according to different filling materials and the interaction between the roller and the soil.

### 6.3. Compound Model

Combining the respective advantages of the vibration compaction model and the AI-based prediction model, some scholars have carried out research on compound models. For improving the intelligent level of subgrade and pavement rolling compaction technology, Lin and Wang [44] established a subgrade and pavement intelligent rolling compaction model, which consists of a viscoelastic–plastic vibration compaction model and an intelligent prediction model of compaction parameters. Prokopev et al. [254] proposed a concept “cyber-physical road construction system” for continuous control compaction of the asphalt mixture. In the hierarchical “study” layer, the results of measurements and forecasts are determined, which are made available to interested specialists, and are used for theoretical analysis using a mathematical model of the object for further adjustment of optimal operating modes. Based on the vibration compaction process model and the compaction quality comprehensive evaluation method, An et al. [255] put forward a dynamic optimization method of compaction process for the rockfill materials, and realized the dynamic optimization of compaction parameters considering the overall optimal solution. Zhang et al. [17,256,257] constructed an intelligent compaction control theory and an intelligent rolling compaction method for compaction quality control and assurance of earth–rock dams, which has certain application potential in the construction of highways, railways, and airports. The preliminary exploration of the compound model in the QC/QA of earthwork shows that it can give full play to the advantages of the vibration compaction model and the prediction model based on AI. The compound model can intelligently adjust the optimal compaction parameters according to the compaction situation and shows great potential in realizing the intelligentization of QC/QA of earthwork. However, it is still less used in earthwork of roads, railways, airports, dams, and embankments.

### 6.4. Intelligent Control Compaction Systems

**Figure 13:**
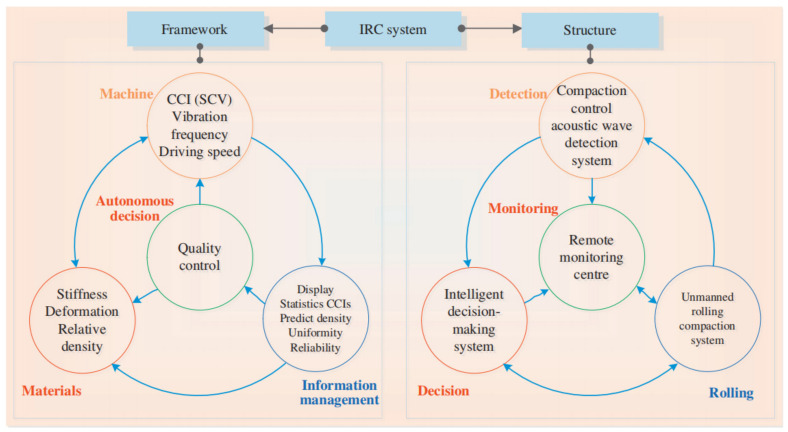
Framework and structure of the IRC system (reprinted with permission from Reference [17], copyright Elsevier, 2020).

Currently, several intelligent control compaction systems (see Table 5) have been developed for the compaction quality control and assurance of earthwork. These systems are all implemented under the framework of machine-material-information-machine intelligent decision-making, and the control criteria for intelligent decision-making are different. Combining the knowledge from the autonomous vehicles area and intelligent asphalt analyzing, Botev and Azidhak [46] proposed a new autonomous compactor (AC) concept. The AC mainly consists of decision systems, IACA, internal communication within the AC system, and autonomous driving. Utilizing prediction model based on AI, Luo and Bi [47] invented an intelligent vibratory roller control system (IVRCS) with unmanned driving characteristics for earthwork. The IVRCS could learn independently and give a reasonable rolling construction plan. To effectively control the filling construction quality of subgrade and pavement, Lin and Wang [44] introduced the concept of an intelligent rolling compaction system for subgrade and pavement, which can realize the dynamic optimization of the compaction process based on the vibration compaction model or the prediction model based on AI, and then provide optimized rolling construction parameters. The intelligent control compaction system based on the compound model has also made some progress. Using the proposed system integration method based on a three-layer structure, Zhang et al. [17] achieved an intelligent rolling compaction system (see Figure 13), which is mainly composed of the continuous compaction control acoustic wave detection system, intelligent decision-making system, unmanned rolling compaction system, and real-time remote monitoring center.

## 7. Conclusions and Future Directions

The essay summarizes the state-of-the-art of the QC/QA methods for earthwork compaction in roads, railways, airports, dams, and embankment construction. In accordance with the technical framework and level, the QC/QA methods are divided into conventional compaction methods, digital rolling compaction methods, automatic rolling compaction methods, and intelligent control compaction methods. It can be found that most of the research efforts focus on: (1) sampling point detection; (2) compaction parameter monitoring and continuous detection methods; (3) automatic driving technology, path planning, and 3D navigation; (4) adopting vibration dynamic model, AI, BIM, data mining, and intelligent control to accelerate the improvement of intelligent level of QC/QA. Based on the above research, various problems existing in conventional QC/QA methods have been partially resolved to achieve more effective compaction quality control and construction efficiency improvement (see Table 6).

However, the field is still far from mature, and quite a few challenges and limitations need further investigation and exploration:

- In terms of digital rolling compaction methods, a comprehensive CCI measurement system considering the uncertainty is needed for single-layer analysis; a simple and realistic mathematical representation of the complex compaction dynamics is also required; real-time calculation and analysis of multi-source heterogeneous data is also an important research direction; standardized application process and cost-benefit assessment in the context of the full life cycle are necessary to be established; improving the utilization level of data in the construction stage and integrating the type of method into the on-site project management architecture more reasonably is also a topic worth studying.
- As far as automatic rolling compaction methods are concerned, the biggest challenges causing slow adoption of methods have been identified as: lack of targeted specifications, unstandardized construction procedures, multi-machine collaborative rolling, adaptive path planning issues, scheduling, and management issues are the research priorities that need to be focused on the next step.
- For intelligent control compaction methods, specifications and construction procedures remain to be gradually formulated and standardized; the effective improvement of computing power and data management level is also an inevitable development trend; the real-time data transmission awaits further optimization. In addition, other directions are gradually attracting the attention of relevant researchers, such as improving visco-elastoplasticity, convergent use of new technologies (BIM, data mining, intelligent control, deep learning, et al.), design and development of expert systems, multi-agent systems and other intelligent control compaction systems, fusion application of pluralistic control thought, and intelligent control theory.

## Figures and Tables

**Table 1:** Related work contributions and comparison (√ is involved).

References	Parameter Type			System	Contribution
	Material Properties	Mechanical Parameters	Construction Parameters		
[97]	-	√	√	CDS	Compaction documentation system for unbound aggregates
[98]	-	-	√	CIRCOM	Computer integrated road construction of compaction
[99]	-	√	-	MSEEF	Monitoring system for the eccentric excitation force
[100]	-	√	-	CIS	Monitor the three-dimensional vibration
[101]	√	-	√	ACRM	Automatic control and monitoring for truck watering
[102]	-	√	√	MRCM	Cyber-physical monitoring for multi-roller compaction
[103]	-	√	√	CQMS	Theory and mathematical model of CQMS based on PCT
[104]	-	-	√	GWIMS	Georobot/WLAN-based intelligent monitoring
[105]	-	-	√	RCQSS	Monitoring the number of compaction times
[106]	-	√	√	CCMS	Continuous compaction monitoring system based BDS
[107]	-	-	√	CEGPS	Monitoring field lift thickness
[108]	-	√	√	CQMCS	Monitoring and control of compaction quality
[109]	-	√	√	RDCQMS	GPS-based monitoring for construction quality

**Table 2:** Introduction of continuous detection methods and CCIs (√ is involved).

Method		CCI	Application Scenarios					Related Research
			Road	Railway	Airport	Dam	Embankment	
Acceleration		CMV	√	√	√	√	√	[1,16,23,25,37,97,112,127,129,133,147,149,151,161,162,163,164,167,168,169,170,171,181]
		RMV	√	-	-	√	√	[112,118,162,172]
		CCV	√	-	-	√	-	[2,133,147,172]
		CV	√	-		√	-	[24,91,122,124,125,130,172]
		CF	-	-	-	√	-	[131]
		$A_{p}$	√	-	-	-	-	[119]
		OMV	√	-	-	-	-	[138]
		AA	√	-	-	-	-	[147]
		$F_{t}$	√	-	√	-	-	[166]
		THD	-	-	-	√	-	[100,123,172,175]
GPR			√	√	√	√	√	[4,113,139,140,152,173,174]
SW	P	$V_{p}$	√	-	√	√	-	[117,142,155,177]
	S	$V_{s}$	√	-	√	√	-	[141,142,159,177]
	Surface		√	√	√	√	√	[6,120,128,143,154,156,157,158,173,174,176,178]
Force		VCV	√	√	-	-	√	[12,34,126,132,133]
		$F_{s}^{'}$	-	-	-	√	-	[125]
Deformation		$k_{s}$	√	-	√	-	-	[30,99,126,133,161]
		$E_{vib}$	√	√	√	-	-	[111,144,145,146,161]
		$M_{i}$	√	-	-	-	-	[114,135,136,137]
Energy		MDP	√	-	-	-	√	[1,23,25,147,148,149,150,151,161,162,163,182]
		Omega	-	-	√	-	-	[2]
		E	√	-	-	√	-	[16,123]
		DMV	-	-	√	-	-	[121]
		CEV	-	√	-	-	-	[168]
FBG			√	-	-	-	-	[165]
Acoustic wave		SCV	-	-	-	√	-	[10,17,36]
ER			√	-	-		√	[116,179,180]
Other		CSD	√	-	-	√	-	[134]
		NCI	-	-	-	-	-	[153]

**Table 3:** Comparison of compaction quality assessment models and methods (√ is involved).

References	Scenarios	Models	Indexes	Methods	Real-Time
[183]	Road	SLR/MLR	Compactness/ Deflection/CMV		
[26]	Railway	SLR	CMV	Combination of δ and R	
[159]		SLR	Cone resistance		
[131]	Dam	SLR/SNR	CF/CMV		
[24]	Dam	SLR/SNR/MNR	Dry density/ Compactness	Geostatistics with CV	
[10]	Dam	SLR/MLR/MNR	Dry density	Geostatistics with SCV	
[9]	Dam	MNR	Compactness	Geostatistics-based	
[184]	Dam	SVR with CFA	Compactness	Combination of *i*-AHP and *i*-GAM	√
[185]	Dam	SVR with CFA		SVR with CFA	
[186]	Dam	SBFA-CKSVR	CMV	SBFA-CKSVR	
[187]	Dam	Cloud-fuzzy		Cloud-fuzzy	
[117]	Dam	SLR			
[91]	Dam	MLR	CV		
[188]	Road	SLR/MLR	Compactness	$\mathrm{Combination}\;\mathrm{of}\;C_{V0}$ and E	
[189]	Airport			Equivalent additional stress	√
[190]		SLR/MLR	$\mathrm{Compactness}/E_{vd}$	Geostatistics with CMV or VCV	
[191]	Dam	CDD		CDD-based	
[192]	Road	SLR	CMV		
[122]	Dam	SLR/MLR	Compactness	CV-based	
[123]	Dam	SLR/MLR/MNR	Dry density	Combination of E and THD	
[193]	Dam	B-ELM	Compactness	B-ELM	√
[124]	Road	SLR	Compactness	Geostatistics with CV	
[194]	Dam	Dual coupled	Dry density	Coupled with dry density and reliability	
[37]	Dam	RBF	Relative density		
[195]	Dam	MNR	Compactness		√
[129]	Dam	Fuzzy	CV	Fuzzy evaluation-based D-S	
[196]	Dam	ANN	Compactness/Dry density	Based-ANN	
[197]	Dam	KM+AC-BFA+FL	Compactness		√

**Table 4:** Automatic rolling compaction methods and unmanned rolling compaction systems (√ is involved).

Author	Contribution	PP	OA	CR
Sun [206]	Automatic control devices and rolling driving methods	√	-	-
Yao et al. [7]	HEMS, mainly including optimal path algorithm and unmanned vehicle control	√	-	-
Yao et al. [207]	Accurate trajectory tracking for self-driving vibratory roller	√	-	-
Song and Zhang [208]	A simulation model build based on the pure pursuit algorithm	√	-	-
Zhang et al. [209]	Optimal path planning of impact roller	√	-	-
Husemann et al. [210]	The evaluation of the impact of different road compaction strategie	√	√	√
Yang et al. [40]	A novel and effective path tracking control of articulated road roller	√	-	-
Song and Xie [211]	A composite disturbance rejection for the path-following control of rollers	√	-	-
Fang et al. [39]	A path following control model for an unmanned vibratory roller	√	-	-
Zhang et al. [17]	Unmanned rolling compaction system, including an unmanned roller, RTK-GPS system, wireless communication system, and remote monitoring center	√	√	√
Huang et al. [213]	Autonomous construction system for an unmanned vibratory roller	√	-	-
Chen et al. [41]	An improved technology for unmanned driving	√	√	-
Shi et al. [42]	Unmanned roller group collaborative complete coverage path planning	√	√	√
Shi [43]	Unmanned rolling dam construction technology of high arch dams	√	-	-
Bian et al. [214]	Path following a control method based on fuzzy algorithm	√	-	-
Zou et al. [215]	A method of obstacle detection based on D-S evidence theory	√	√	-

**Table 5:** Introduction of intelligent control compaction systems (√ is involved).

System	Vibration Compaction Model	Prediction Model Based on AI	Compound Model
AC [46]	-	√	-
IVRCS [47]	-	√	-
IRCSP [44]	√	√	-
IRC [17]	√	√	√

**Table 6:** Comparison of advantages of different QC/QA methods (√ is involved).

	Solution	Conventional Compaction Methods	Digital Rolling Compaction Methods	Automatic Rolling Compaction Methods	Intelligent Control Compaction Methods
Problem					
The number of compaction times is not up to standard		-	√	√	√
Rolling omission, cross rolling, and hypervelocity		-	-	√	√
Quality detection of the entire working area		-	√	-	√
Feedback control is not accurate and timely		-	-	-	√

## Data Availability

Not applicable.

## Abbreviations

The following abbreviations are used in this manuscript:

CMV	Compaction Meter Value
RMV	Resonant Meter Value
CCV	Compaction Control Value
CV	Compaction Value
CF	Crest Factor Value
$A_{p}$	Acceleration Peak Value
OMV	Oscillometer Value
AA	Acceleration Amplitude
$F_{t}$	Turbulence Factor
THD	Total Harmonic Distortion
$V_{p}$	P-wave Velocity
$V_{s}$	S-wave Velocity
VCV	Vibratory Compaction Value
$F_{s}^{'}$	Improved Index of Ground Reaction Force Index
$k_{s}$	Material Stiffness
$E_{vib}$	Geomaterial Dynamic Modulus
$M_{i}$	Intelligent Compaction Analyzer Modulus
MDP	Machine Drive Power
Omega	Energy Transmitted to the Soil
E	Compaction Power Per Unit Volume
DMV	Dissipation Measured Value
CEV	Compaction Energy Value
SCV	Sound Compaction Value
CSD	Contact Stress Distribution
NCI	Normalized Compaction Indicator
